# Proton radiotherapy for chest wall and regional lymphatic radiation; dose comparisons and treatment delivery

**DOI:** 10.1186/1748-717X-8-71

**Published:** 2013-03-24

**Authors:** Shannon M MacDonald, Rachel Jimenez, Peter Paetzold, Judith Adams, Jonathan Beatty, Thomas F DeLaney, Hanne Kooy, Alphonse G Taghian, Hsiao-Ming Lu

**Affiliations:** 1 Department of Radiation Oncology, Massachusetts General Hospital, Harvard Medical School, Cox 340, 100 Blossom Street, Boston, MA, 02114, USA

**Keywords:** Breast cancer, Treatment planning, Proton beam radiation

## Abstract

**Purpose:**

The delivery of post-mastectomy radiation therapy (PMRT) can be challenging for patients with left sided breast cancer that have undergone mastectomy. This study investigates the use of protons for PMRT in selected patients with unfavorable cardiac anatomy. We also report the first clinical application of protons for these patients.

**Methods and materials:**

Eleven patients were planned with protons, partially wide tangent photon fields (PWTF), and photon/electron (P/E) fields. Plans were generated with the goal of achieving 95% coverage of target volumes while maximally sparing cardiac and pulmonary structures. In addition, we report on two patients with unfavorable cardiac anatomy and IMN involvement that were treated with a mix of proton and standard radiation.

**Results:**

PWTF, P/E, and proton plans were generated and compared. Reasonable target volume coverage was achieved with PWTF and P/E fields, but proton therapy achieved superior coverage with a more homogeneous plan. Substantial cardiac and pulmonary sparing was achieved with proton therapy as compared to PWTF and P/E. In the two clinical cases, the delivery of proton radiation with a 7.2 to 9 Gy photon and electron component was feasible and well tolerated. Akimbo positioning was necessary for gantry clearance for one patient; the other was treated on a breast board with standard positioning (arms above her head). LAO field arrangement was used for both patients. Erythema and fatigue were the only noted side effects.

**Conclusions:**

Proton RT enables delivery of radiation to the chest wall and regional lymphatics, including the IMN, without compromise of coverage and with improved sparing of surrounding normal structures. This treatment is feasible, however, optimal patient set up may vary and field size is limited without multiple fields/matching.

## Introduction

Post-mastectomy radiation therapy (RT) improves disease free survival (DFS) and overall survival (OS) for locally advanced breast cancer (LABC) [1]. Unfortunately, radiation to the chest wall and regional lymphatics carries risks of cardiopulmonary and other toxicities [2]. Although modern techniques minimize high dose radiation to avoidance structures, some patients with advanced disease and/or unfavorable anatomy still present a challenge for radiation planning and compromises in target coverage or desired sparing of cardiopulmonary structures is often necessary.

Proton radiation is a form of particle radiation capable of delivering therapeutic radiation with complete sparing of tissues beyond the target. Mainly due to patient capacity limits in the relatively small number of clinical proton facilities, the clinical use of protons has been limited to tumors requiring high doses or to those in close proximity to critical structures (e.g. brain, spinal cord). Despite substantial capital and operational costs, several proton facilities have recently opened or are in planning or construction phases. Furthermore, several companies are actively researching more efficient, smaller, and less expensive equipment for proton therapy delivery. Given the increasing availability of proton radiation in both academic and private sectors, it is critical to evaluate potential benefits and techniques for the treatment of additional malignancies, such as breast cancer, for which the defined range of the proton might reduce normal tissue radiation dose, allow concurrent chemotherapy or some combination thereof for an improvement in patient outcome. Comparative planning studies hypothesize that protons will provide a decrease in acute and late cardiopulmonary toxicities for patients requiring RT for advanced or left sided breast cancer, but no clinical experience has been reported to date for this group of patients [3-5]. We report dosimetric comparisons for eleven patients with left sided breast cancer requiring post-mastectomy radiation therapy (PMRT) planned with 3D CRT, partially wide tangent fields (PWTF), and a mixed photon electron (P/E) technique to compare target coverage and conformality across these treatment modalities. We also report on the clinical technique and feasibility for two patients treated with a combination of proton and photon radiation for LABC.

## Methods and materials

### Dosimetric comparisons

For eleven representative cases, we compared PWTF, mixed P/E technique, and 3D-conformal, passively scattered proton beam radiation. Plans were performed with the attempt to achieve 95% coverage of target volumes (chest wall, internal mammary lymph nodes (IMN), supraclavicular lymph nodes (SCV), and axilla) while maximally sparing cardiac and pulmonary structures. Four patients were planned for treatment to the chest wall and IMN only. Seven were planned for chest wall, IMN, SCV, and axillary apex or full axilla. Priority was given to target volume coverage. Field-in-field technique was used in PWTF and P/E plans to minimize hot spots. Compromise of target coverage was not allowed to minimize hot spots or spare cardiopulmonary structures. Standard proton planning was performed with XiO planning software (CMS Inc., St Louis, Missouri). The XXX Proton Therapy Center provides a rotational gantry system and maximum proton beam energy of 235-MeV. The CTV prescription for all dosimetric comparisons was 50 Gy (RBE).

### Patients

Two women with locally advanced/inflammatory breast cancer were referred to the Francis H. Burr Proton Facility for proton radiation after difficulty in radiation planning with conventional techniques. Dedicated planning with Computed Tomography (CT) scans was obtained. Patients were immobilized, with a custom Civco™ breast board in the supine position, one with left arm akimbo due to limited arm mobility and to allow for clearance of the gantry and one with both arms above her head. The chest wall, regional lymphatics (SCV, level 1, 2, 3 axilla, and IMN), and organs at risk were contoured according to RTOG guidelines by a radiation oncologist [6]. CTV was defined as a combined volume of all target structures. An additional margin of 8 to 10 mm was added around the CTV to account for both lateral beam penumbra and PTV together. Correspondingly, 3-5 mm was used for smearing radius in the design of compensators. Three and half percent of the maximum beam range was used to account for range uncertainty. Customized brass apertures and Lucite compensators were fabricated on-site by computerized milling machines interfaced with the treatment planning system for each patient. Daily positioning was achieved based on bony landmarks with diagnostic quality orthogonal x-rays compared to Digitally Reconstructed Radiographs (DRRs). A computer program assisted the therapists in making patient couch shifts as needed with six degrees of freedom to more exactly align the patients [7].

A dose of 50.4 Gy (RBE) was prescribed, employing the relative biological effectiveness (RBE) value of 1.1 [8]. One patient received an additional IMN boost of 5.4 Gy (RBE) to deliver a total dose 55.8 Gy (RBE) to involved IMNs. Field arrangement chosen to minimize dose to critical structures while maximizing target coverage was a single field LAO or matched LAO fields (chest wall and “SCV” field). Feathering was employed at match line.

## Results

**Figure 1:**
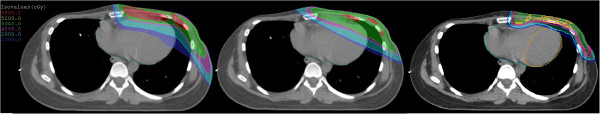
Axial images at the level of the heart for photon/electron plan (left), partially wide tangent fields (middle), and protons (right).

**Figure 2:**
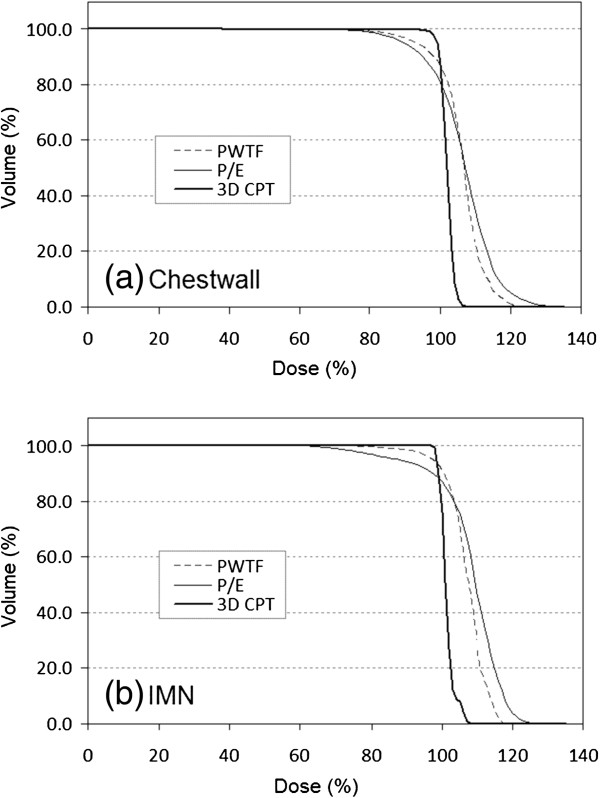
Dose volume histograms for chest wall (a) and internal mammary nodes (b) averaged over the patients for the three treatment techniques PWTF (dashed), P/E (thin solid) and 3D CPT (thick solid).

Of the eleven patients used for dosimetric comparison, all had undergone mastectomy without breast reconstruction and had left sided breast cancer. 3D CPT, PWTF and P/E plans were generated and compared. All plans attempted to deliver a target dose of 50 Gy or Gy (RBE) to 95% of the CTV (chest wall and regional lymphatics; all patients had chest wall and IMN targeted +/- level 1, 2, 3 axillary lymph nodes, and SCV) while sparing cardiac and pulmonary structures. Comparable tumor volume coverage was achieved with PWTF and P/E fields, but proton therapy achieved superior coverage with a noticeably more homogeneous plan and decreased maximum % dose or “hot spot”. (Table 1, Figures 1, 2, &3). Substantial normal tissue sparing was seen with the proton therapy as compared to PWTF and P/E (Table 1, Figures 1 &4). The volume of heart receiving 20 Gy or Gy (RBE) was 12%, 12.4%, and 1.6% for PWTF, P/E, and 3D CPT, respectively. The average V20 for ipsilateral lung was 25.3%, 21.7%, and 16.2% respectively for PWTF, P/E, and 3D-CPT. Figure 5 demonstrates the improved cardiac and pulmonary sparing achieved with protons. Coverage of the IMN with 95% was easily achieved with proton plans, but was not easily achieved with all P/E plans due to the depth of the upper IMN in some patients (Figure 2).

**Table 1:** Average volumes (%) of critical organs and targets at specific dose levels (Gy RBE) for the three treatment techniques, PWTF, P/E, and 3D CPT, with the range of values shown in parentheses

**Vol (%)**	**Dose levels**	**PWTF**	**P/E**	**3D CPT**
Heart	V5Gy	20.9 (14.1–29.2)	35.6 (28.6–45.7)	4.1 (2.6–7.6)
	V10Gy	14.9 (8.9–21.9)	22.7 (15.2–31.8)	2.8 (1.6–6.1)
	V20Gy	12.0 (6.6–18.4)	12.4 (6.3–18.9)	1.6 (0.6–4.5)
	V45Gy	6.9 (2.8–12.4)	2.2 (0.1–5.5)	0.3 (0.0–1.6)
Lung	V5Gy	33.2 (12.1–50.0)	46.3 (28.1–70.4)	25.2 (9.8–36.2)
	V10Gy	28.5 (10.0–42.5)	32.2 (17.0–55.7)	21.3 (8.0–31.8)
	V20Gy	25.3 (8.7–36.3)	21.7 (11.8–39.5)	16.2 (5.9–25.9)
Chestwall	V45Gy	96.6 (92.6–99.4)	94.9 (88.8–99.0)	99.9 (98.7–100.0)
	V47.5Gy	93.6 (87.3–97.7)	90.0 (81.6–95.8)	99.6 (98.1–100.0)
IMN	V45Gy	98.1 (90.6–100.0)	94.0 (84.3–100.0)	100.0 (99.7–100.0)
	V47.5Gy	96.6 (86.5–100.0)	91.8 (80.5–99.7)	99.9 (99.0–100.0)
Level 1	V45Gy	90.4 (69.2–100.0)	93.8 (70.2–100.0)	99.9 (99.7–100.0)
	V47.5Gy	85.7 (52.7–100.0)	91.4 (60.5–100.0)	99.6 (98.8–100.0)
Level 2	V45Gy	95.0 (76.3–100.0)	93.5 (81.7–100.0)	100.0 (100.0–100.0)
	V47.5Gy	89.9 (56.4–99.8)	90.7 (70.5–100.0)	100.0 (100.0–100.0)
Level 3	V45Gy	97.6 (92.2–100.0)	85.5 (73.5–99.2)	100.0 (99.9–100.0)
	V47.5Gy	93.7 (82.2–100.0)	82.4 (71.0–98.5)	96.8 (92.8–100.0)
SCV	V45Gy	98.2 (93.1–100.0)	98.6 (97.2–100.0)	98.6 (92.0–100.0)
	V47.5Gy	95.9 (91.8–100.0)	95.7 (87.3–100.0)	95.0 (89.0–100.0)

**Figure 3:**
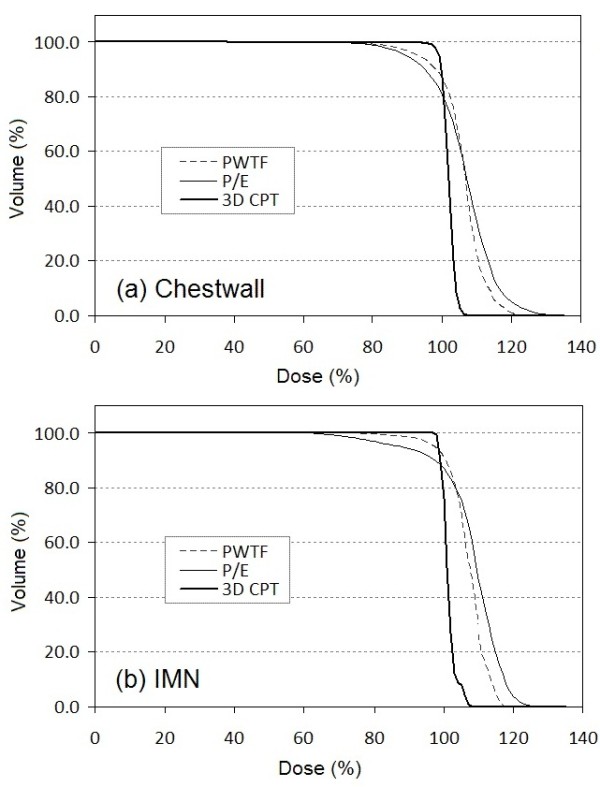
Dose volume histograms for nodal target volumes, Level I (a), II (b), III (c) and SCV, averaged over the patients for the three treatment techniques PWTF (dashed), P/E (thin solid) and 3D CPT (thick solid).

**Figure 4:**
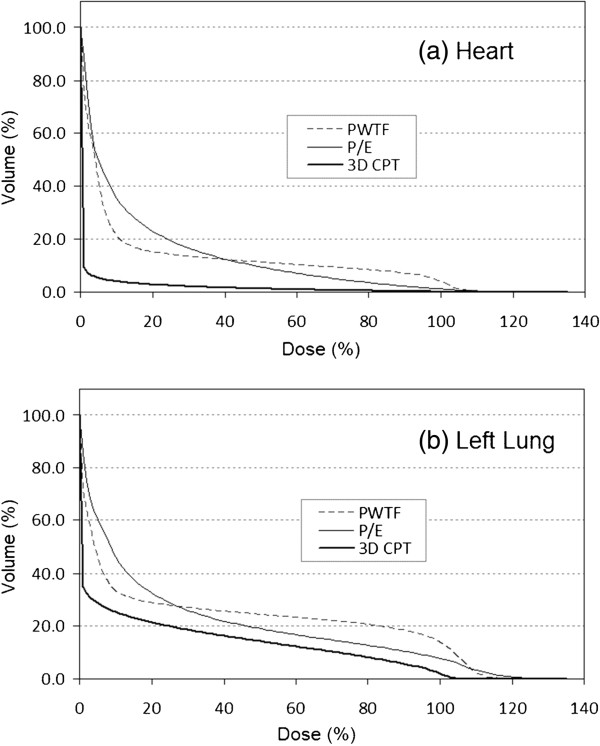
Dose volume histograms for heart (a) and left lung (b) over the patients for the three treatment techniques PWTF (dashed), P/E (thin solid) and 3D CPT (thick solid).

**Figure 5:**
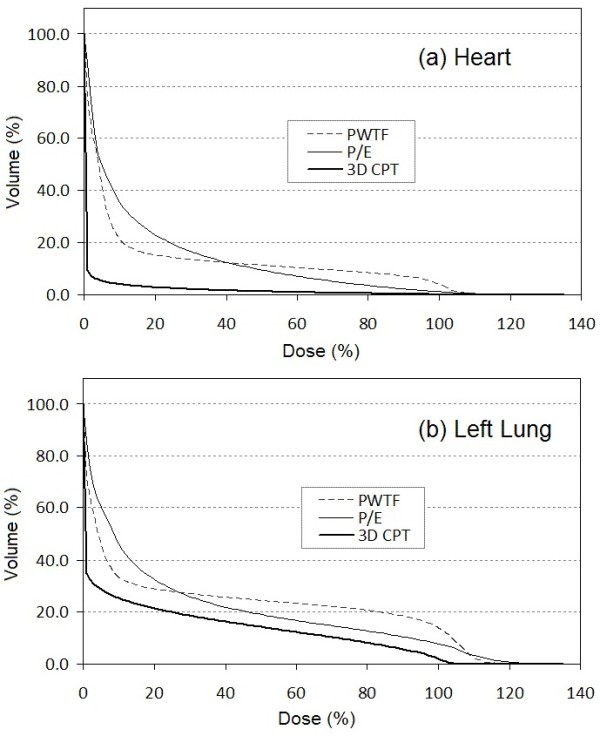
**Skin reactions during and up to one year after proton radiation treatment for 2 patients that received proton radiation. a)** Skin reaction for a patient that received proton radiation to the chest wall and regional lymphatics to a total dose of 50.4 Gy (RBE) with 9 Gy of photon/electron treatment. **b)** Patient that received 50.4 Gy (RBE) to chest wall and regional lymphatics followed by boost to total 55.8 Gy (RBE) to a portion of the IMN chain with 7.2 Gy of photon/electron treatment.

Of the two patients treated with proton radiation, technical delivery was feasible and without complication Four or five of twenty-eight to thirty-one fractions (7.2–9 Gy of 50.4-55.8 Gy (RBE) were delivered with photon/electron plans for the two patients treated. The course of radiation was tolerated well. Grade 2 erythema and fatigue were the only noted acute side effects. Figure 1 depicts skin toxicities during and up to 1 year after treatment. The two patients did not experience radiation pneumonitis, dysphagia, rib fracture, lymphedema, brachial plexopathy, or any unanticipated side effect of treatment at 6 months to 1 year following radiation. Both are without evidence of disease recurrence.

## Discussion

Earlier radiation trials reported an increase in morbidity and mortality due to cardiac disease, predominantly in patients treated for left sided breast cancer. Some authors have suggested that increased mortality from ischemic heart disease was the reason for the absence of a survival benefit for these patients [9]. More recent post-mastectomy studies utilizing modern techniques have demonstrated survival benefit without increase in cardiac mortality to date, but longer follow up is necessary. SPECT and strain echocardiogram results report changes in cardiac tissue with doses as low as 3 Gy, but whether or not these early cardiovascular changes can be used as a surrogate for late cardiac outcomes has not yet been determined [2,10,11]. The increased use of cardiotoxic chemotherapy over the past several years adds yet another confounding factor to determining the effect of radiation therapy on cardiac outcomes and it is not yet known how radiation in the setting of these agents with impact late cardiovascular outcomes [12,13]. Therefore, maximal cardiac sparing achieved through proton therapy has the potential to decrease this risk by decreasing mean heart dose as well as volume receiving 40 Gy and 25 Gy (Table 1). It is predicted that for select patients, protons may offer a reduction in late cardiac morbidity that may ultimately prove to be cost effective [14]. For some patients, breath hold techniques or the addition of a heart block may provide adequate sparing of the heart without compromising chest wall and IMN coverage. For patients that have lower inner quadrant primary tumors, LVI, or inadequate displacement or the heart with breath hold, protons may provide an alternative, albeit more expensive, treatment.

Radiation pneumonitis is a sub-acute side effect reported in approximately 1-5% of patients treated for breast cancer without concurrent chemotherapy; higher rates are seen with concurrent Taxol [15]. Delivery of conventional chest wall and regional lymphatic RT may result in delivery of 20 Gy to 20–40% of the lung. In addition, pneumonitis rates have been shown to increase when large volumes of lung receive low dose irradiation (5 Gy or 10 Gy) in lung cancer patients [16]. Although the meaning of low dose lung irradiation for patients with breast cancer is less clear, the use of techniques increasing the total volume of tissue receiving radiation (e.g. IMRT, electrons) heightens concern for radiation-induced malignancies, particularly for young women. Protons are capable of both reducing high doses of radiation and avoiding exposure of uninvolved tissues to low dose radiation exposure. (Figure 4, & Table 1).

Inclusion of the IMN for LABC remains quite controversial. The majority of PMRT trials demonstrating benefit included treatment of the IMN [1,17]. In addition, Whelan, et al recently reported a DFS benefit and trend in improved OS for women treated to the regional lymphatics including the IMN on the National Cancer Institute of Canada (NCIC) MA-20 trial [18]. These data indicate a benefit for inclusion of the IMN. Also, with the use of positron emission tomography (PET) and high-resolution computed tomography (CT) scans, suspicious IMN are sometimes detected, obliging inclusion of the IMN in the radiation field [19]. The rationale for excluding IMN treatment when delivering PMRT is that the risks of increased cardiopulmonary toxicity negate any potential benefit in DFS. Proton therapy allows for treatment and superior coverage of these sometimes deep-seated lymph nodes with minimal cardiopulmonary dose perhaps tipping the risk-benefit ratio in favor of IMN inclusion.

One concern in delivering passively scattered proton radiation to either the breast or the chest wall is the increased dose to the skin. Delivery of proton radiation with a small photon or electron component (7.2–9 Gy of 50.4 to 55.8 Gy (RBE) was feasible and well tolerated. Skin toxicity was both acceptable and within the range of what would be expected for standard treatment. For PMRT, the skin is considered a target, and bolus or electrons are often used to increase dose to the skin. In addition, although cosmesis is an important outcome, cardiopulmonary sparing and target coverage are the primary goals of treatment for these women with locally advanced breast cancer. For these reasons, we believe that this is an ideal population of patients to explore the potential benefits and risks of proton radiation specific to breast cancer.

Another frequently mentioned concern regarding the use of proton radiation is cost. Although the dosimetry delivered by proton therapy is clearly superior to that of standard RT, clearly superior clinical outcomes are *also* necessary to justify the higher cost of proton therapy. Lundkvist et al, performed a cost analysis indicating that for carefully selected patients, specifically where cardiac sparing was at issue, proton therapy could be cost-effective [14]. If the cost of proton therapy eventually decreases, and patients are carefully selected, proton therapy may prove cost-effective for selected subgroups of breast cancer patients.

## Conclusions

The rationale for the use of proton radiation for the treatment of breast cancer is to decrease late toxicity by reducing the dose delivered to cardiopulmonary structures without compromising desired target volume coverage. This study demonstrates the dosimetric advantages of protons over PWTF and P/E technique for representative patients with left sided breast cancer requiring PMRT and reports on the first clinical use of proton radiation for post-mastectomy patients. We conclude that protons may be of benefit for a select population of patients with locally advanced breast cancer. We are now actively accruing patients to a clinical trial examining the feasibility of protons for PMRT (without a photon or electron component) in the setting of complex anatomy due to unfavorable cardiac anatomy or breast prostheses.

## Consent

Written informed consent was obtained from the patient for publication of this report and any accompanying images.

## Competing interests

The authors declare that they have no competing interests.

## Authors’ contributions

All authors have read and approved the manuscript and agree to its submission. This manuscript has not been previously published. All authors report no conflicts of interest.
